# Association of stress hyperglycemia ratio with left ventricular function and microvascular obstruction in patients with ST-segment elevation myocardial infarction: a 3.0 T cardiac magnetic resonance study

**DOI:** 10.1186/s12933-024-02271-6

**Published:** 2024-05-27

**Authors:** Kairui Bo, Weibo Li, Hongkai Zhang, Yan Wang, Zhen Zhou, Yifeng Gao, Zhonghua Sun, Jianxiu Lian, Hui Wang, Lei Xu

**Affiliations:** 1 Department of Radiology, Beijing Anzhen Hospital, Capital Medical University, No. 2 Anzhen Rd, Chaoyang District, Beijing, China; 2 Department of Emergency Medicine, Beijing Friendship Hospital, Capital Medical University, Beijing, China; 3 Discipline of Medical Radiation Science, Curtin Medical School, Curtin University, Perth, WA 6845 Australia; 4 Philips Healthcare, Beijing, China

**Keywords:** Stress hyperglycemia ratio, Acute ST-segment elevation myocardial infarction, Cardiovascular magnetic resonance, Global peak strain, Microvascular obstruction

## Abstract

**Background:**

Stress hyperglycemia, which is associated with poor prognosis in patients with acute myocardial infarction (AMI), can be determined using the stress hyperglycemia ratio (SHR). Impaired left ventricular function and microvascular obstruction (MVO) diagnosed using cardiac magnetic resonance (CMR) have also been proven to be linked to poor prognosis in patients with AMI and aid in risk stratification. However, there have been no studies on the correlation between fasting SHR and left ventricular function and MVO in patients with acute ST-segment elevation myocardial infarction (ASTEMI). Therefore, this study aimed to investigate the additive effect of fasting SHR on left ventricular function and global deformation in patients with ASTEMI and to explore the association between fasting SHR and MVO.

**Methods:**

Consecutive patients who underwent CMR at index admission (3–7 days) after primary percutaneous coronary intervention (PPCI) were enrolled in this study. Basic clinical, biochemical, and CMR data were obtained and compared among all patients grouped by fasting SHR tertiles: SHR1: SHR < 0.85; SHR2: 0.85 ≤ SHR < 1.01; and SHR3: SHR ≥ 1.01. Spearman’s rho (r) was used to assess the relationship between fasting SHR and left ventricular function, myocardial strain, and the extent of MVO. Multivariable linear regression analysis was performed to evaluate the determinants of left ventricular function and myocardial strain impairment in all patients with AMI. Univariable and multivariable regression analyses were performed to investigate the correlation between fasting SHR and the presence and extent of MVO in patients with AMI and those with AMI and diabetes mellitus (DM).

**Results:**

A total of 357 patients with ASTEMI were enrolled in this study. Left ventricular ejection fraction (LVEF) and left ventricular global function index (LVGFI) were significantly lower in SHR2 and SHR3 than in SHR1. Compared with SHR1 and SHR2 groups, left ventricular strain was lower in SHR3, as evidenced by global radial (GRS), global circumferential (GCS), and global longitudinal (GLS) strains. Fasting SHR were negatively correlated with LVEF, LVGFI, and GRS (r = − 0.252; r = − 0.261; and r = − 0.245; all *P*<0.001) and positively correlated with GCS (r = 0.221) and GLS (r = 0.249; all *P* <0.001). Multivariable linear regression analysis showed that fasting SHR was an independent determinant of impaired LVEF, LVGFI, GRS, and GLS. Furthermore, multivariable regression analysis after adjusting for covariates signified that fasting SHR was associated with the presence and extent of MVO in patients with AMI and those with AMI and DM.

**Conclusion:**

Fasting SHR in patients with ASTEMI successfully treated using PPCI is independently associated with impaired cardiac function and MVO. In patients with AMI and DM, fasting SHR is an independent determinant of the presence and extent of MVO.

**Supplementary Information:**

The online version contains supplementary material available at 10.1186/s12933-024-02271-6.

## Introduction

Stress hyperglycemia (SH) refers to a relatively dramatic increase in blood glucose levels owing to many critical illnesses, including acute myocardial infarction (AMI). Increasing evidence suggests that SH may be associated with poor short-term and long-term prognosis in patients with AMI [1–4]. Previous studies have reported that patients with AMI and hyperglycemia at admission have a larger infarct size; higher incidence of microvascular obstruction (MVO), congestive heart failure, and cardiogenic shock; and higher mortality [5–7]. However, the use of admission blood glucose (ABG) level to assess SH ignores the effect of long-term blood glucose levels and may not reflect the true SH situation, especially in patients with AMI and diabetes mellitus (DM). Roberts et al. [8] have devised a stress hyperglycemia ratio (SHR) index to normalize the acute increase in glucose levels in relation to background glycemic status. To date, certain studies have reported poor clinical outcomes in patients with AMI who have a high SHR, and this ratio is expected to be a better predictor of stress-induced hyperglycemia throughout the glycemic profile [9–11]. In fact, conventional SHR calculated from ABG and glycated hemoglobin A1c (HbA1c) may also be influenced by meal times, and Cuiet al. [12] have suggested that fasting SHR is more predictive of in-hospital mortality in patients with AMI than conventional SHR.

**Table 1:** Baseline characteristics of the study population

Variables	Overall (*n* = 357)	SHR < 0.85 (*n* = 121)	0.85 ≤ SHR<1.01 (*n* = 115)	SHR ≥ 1.01 (*n* = 121)	*P* value
Baseline characteristics					
Age, years	56.9 ± 11.2	56.8 ± 11.7	55.4 ± 11.5	58.2 ± 10.2	0.141
Male, n (%)	305 (85.4)	97 (80.2)	101 (87.8)	107 (88.4)	0.129
BMI, kg/m^2^	26.0 ± 3.3	26.2 ± 3.2	26.2 ± 3.3	25.5 ± 3.5	0.472
Systolic blood pressure, mmHg	122.3 ± 17.8	121.7 ± 17.3	121.1 ± 19.5	124.1 ± 16.5	0.294
Diastolic blood pressure, mmHg	76.0 ± 11.6	75.3 ± 11.3	76.0 ± 12.7	76.8 ± 10.8	0.446
Heart rate, bpm	78.3 ± 12.9	76.3 ± 11.7	76.1 ± 12.0	82.5 ± 13.9^bc^	**< 0.001**
Cardiovascular risk factors					
Previous/current smoker, n (%)	233 (65.3)	77 (63.6)	84 (73.0)	72 (59.5)^b^	0.083
Current smoker, n (%)	208 (58.3)	69 (57.0)	73 (63.5)	66 (54.5)	0.359
Hypertension, n (%)	219 (61.3)	75 (62.0)	65 (56.5)	79 (65.3)	0.380
Diabetes, n (%)	132 (37)	46 (38.0)	29 (25.2)	57(47.1)^b^	**0.002**
Dyslipidemia, n (%)	243 (68.1)	83 (68.6)	81 (70.4)	79 (65.3)	0.691
Prior myocardial infarction, n (%)	11 (3.1)	4 (3.3)	3 (2.6)	4 (3.3)	0.939
Previous PCI or CABG, n (%)	19 (5.3)	7 (5.8)	5 (4.3)	7 (5.8)	0.853
Killip class, n (%)					0.606
I	263 (73.7)	92 (76.0)	81 (70.4)	90 (74.4)	
II	83 (23.2)	26 (21.5)	30 (26.1)	27 (22.3)	
III	4 (1.1)	1 (0.8)	2 (1.7)	1 (0.8)	
IV	7 (2.0)	2 (1.7)	2 (1.7)	3 (2.5)	
Blood results					
Blood glucose on admission mmol/L	8.3 (6.9,11.5)	8.0 (6.7,10.0)	7.8 (6.7,10.9)	9.5 (7.5,14.8)^bc^	**< 0.001**
Fasting blood glucose, mmol/L	6.4 (5.5,8.8)	5.5 (5.1,6.2)	6.1 (5.7,7.7)^a^	8.7 (7.1,12.3)^bc^	**< 0.001**
HbA1c, %	6.0 (5.6,7.2)	6.0 (5.7,7.0)	5.8 (5.5,6.9)^a^	6.0 (5.6,7.7)	**0.019**
Fasting SHR	0.9 (0.8,1.1)	0.8 (0.7,0.8)	0.9 (0.9,1.0)^a^	1.1 (1.1,1.3)^bc^	**< 0.001**
CKMB mass, ng/ml	196.5 (86.0,303.0)	142.0 (60.4,252.3)	207.7 (102.9,303.0)^a^	231.5 (111.3,303.0)^c^	**< 0.001**
Myoglobin, ug/L	221.6 (64.0,473.8)	191.1 (60.5,401.9)	259.0 (64.0,520.0)	233.2 (74.0,499.0)	0.186
BNP, pg/ml	178.0 (81.5,316.0)	143.0 (75.5,294.6)	183.0 (97.0,308.0)	202.0 (78.0,328.5)	0.289
Creatinine, umol/L	72.0 (63.8,84.0)	74.1 (64.0,86.3)	72.0 (64.7,83.6)	70.7 (62.7,80.2)	0.421
eGFR, mL/min/1.73 m^2^	97.7 (87.9,107.3)	96.8 (79.4,106.3)	97.9 (89.7,108.3)	99.3 (90.2,107.7)	0.155
Triglycerides, mmol/L	1.5 (1.1,2.1)	1.4 (1.2,2.1)	1.5 (1.1,2.1)	1.4 (1.0,2.0)	0.581
Total cholesterol, mmol/L	4.6 (4.0,5.5)	4.7 (3.9,5.7)	4.6 (4.0,5.6)	4.7 (4.0,5.4)	0.856
HDL cholesterol, mmol/L	1.0 (0.9,1.2))	1.0 (0.8,1.2)	1.0 (0.9,1.2)	1.0 (0.9,1.2)	0.067
LDL cholesterol, mmol/L	3.0 (2.4,3.6)	2.9 (2.4,3.8)	2.9 (2.4,3.6)	3.1 (2.4,3.7)	0.829
High-sensitive CRP, mg/L	5.1 (2.3,11.2)	5.0 (2.3,9.7)	5.2 (2.0,12.3)	5.5 (2.2,12.8)	0.702
Procedures					
Number of diseased arteries, n (%)					0.834
1	145 (40.6)	44 (36.4)	47 (40.9)	54 (44.6)	
2	108 (30.3)	43 (35.5)	36 (31.3)	29 (24.0)	
3	104 (29.1)	34 (28.1)	32 (27.8)	38 (31.4)	
Location of culprit lesion, n (%)					0.181
LAD	189 (52.9)	58 (47.9)	59 (51.3)	72 (59.5)	
LCX	44 (12.3)	15 (12.4)	16 (13.9)	13 (10.7)	
RCA	124 (34.7)	48 (39.7)	40 (34.8)	36 (29.8)	
TIMI flow grade 0/1 pre-PCI, n (%)	276 (77.3)	70 (74.4)	88 (76.5)	98 (81.0)	0.458
TIMI flow grade 3 post-PCI, n (%)	344 (96.4)	116 (95.9)	112 (97.4)	116 (95.9)	0.773
Medications					
Aspirin, n (%)	338 (94.7)	113 (93.4)	110 (95.7)	115 (95.0)	0.724
P2Y12receptor inhibitor, n (%)	350 (98.0)	120 (99.2)	113 (98.3)	117 (96.7)	0.373
β blockers, n (%)	247 (69.2)	82 (67.8)	80 (69.6)	85 (70.2)	0.912
ACEI/ARB, n (%)	199 (55.7)	66 (54.5)	67 (58.3)	64 (54.5)	0.805
Statins, n (%)	334 (93.6)	114 (94.2)	107 (93.0)	113 (93.4)	0.931

**Table 1:** (continued)

Variables	Overall (*n* = 357)	SHR < 0.85 (*n* = 121)	0.85 ≤ SHR<1.01 (*n* = 115)	SHR ≥ 1.01 (*n* = 121)	*P* value
Oral hypoglycemic drugs, n (%)	87 (24.4)	27 (22.3)	29 (25.2)	31 (25.6)	0.425
Insulin, n (%)	44 (12.3)	13 (10.7)	14 (12.2)	17 (14.0)	0.615

*P* values < 0.05 indicate significance (bolded).

SHR: stress hyperglycemia ratio; BMI: body-mass index; PCI: percutaneous coronary intervention; CABG: coronary artery bypass grafting; HbA1c: glycated hemoglobin A1c; CKMB: creatine kinase-myocardial band; BNP: brain natriuretic peptide; eGFR: estimated glomerular filtration rate; HDL: high-density lipoprotein; LDL: low-density lipoprotein; CRP: C-reactive-protein; LAD: left anterior descending; LCX: left circumflex artery; RCA: right coronary artery; TIMI: thrombolysis in myocardial infarction; PCI: percutaneous coronary intervention; ACEI: angiotensin converting enzyme inhibitor; ARB: angiotensin receptor blocker.

^a^*P* < 0.0167 for 0.85 ≤ SHR < 1.01 vs. SHR <  0.85.

^b^*P* < 0.0167 for 0.85 ≤ SHR< 1.01 vs. SHR ≥  1.01.

^c^*P* < 0.0167 for SHR ≥ 1.01 vs. SHR <  0.85.

Cardiac magnetic resonance (CMR) imaging provides comprehensive information on cardiac function, deformation, and myocardial tissue properties. Aspects of left ventricular function, including myocardial strain and presence and extent of MVO, have been shown to be associated with the prognosis in patients with AMI and contribute to risk stratification [13, 14]. However, no studies have so far examined the correlation between fasting SHR and left ventricular function, myocardial strain, and MVO obtained using CMR in patients with acute ST-segment elevation myocardial infarction (ASTEMI). Therefore, this study aimed to investigate the additive effect of fasting SHR on left ventricular function and global deformation in patients with ASTEMI and to explore the association between fasting SHR and CMR-derived MVO after primary percutaneous coronary intervention (PPCI).

**Fig. 1:**
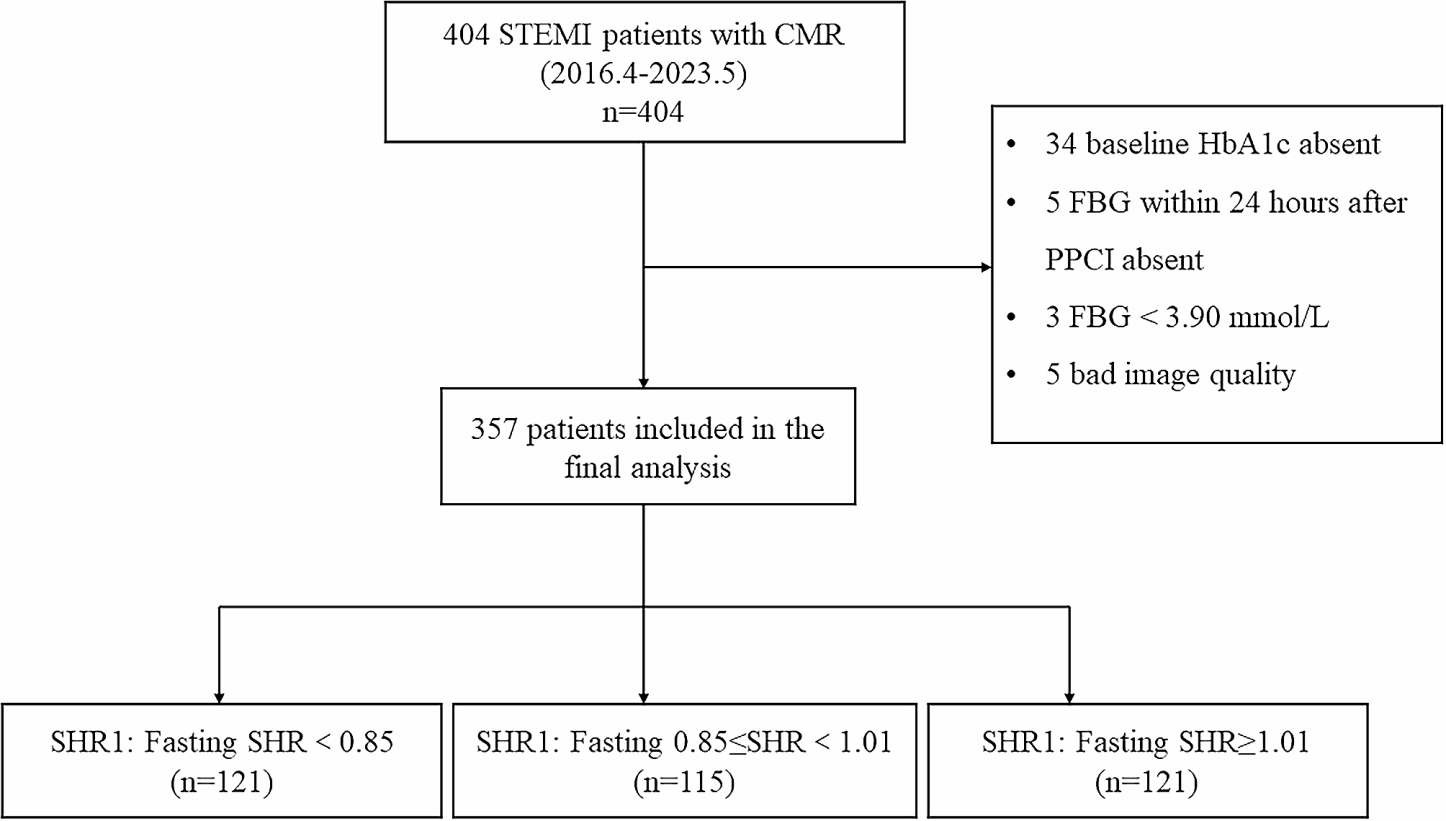
Flow diagram of the patients included in the study.  STEM: ST-segment elevation myocardial infarction; CMR: cardiac magnetic resonance imaging; PPCI: primary percutaneous coronary intervention; HbA1c: glycated hemoglobin A1c; FBG: fasting blood glucose; SHR: stress hyperglycemia ratio

## Methods

### Study population and design

This study was an observational study conducted at Beijing Anzhen Hospital, Capital Medical University. The study protocol conformed to the Declaration of Helsinki and was approved by the Ethics Committee of Anzhen Hospital. We retrospectively enrolled 404 patients with ASTEMI who had completed CMR examinations at Anzhen hospital between April 2016 and May 2023 were enrolled. The inclusion criteria were as follows: (1) The diagnosis of ASTEMI was based on the criteria from the fourth universal definition of myocardial infarction [15]. (2) CMR was performed within 3–7 days after admission for PPCI. The exclusion criteria were as follows: (1) confirmed cardiomyopathy, congenital heart disease, pericardial disease, severe arrhythmia, or valvular disease (2) loss of vital laboratory data; HbA1c and fasting blood glucose (FBG) not obtained within 24 h of PPCI (3) FBG < 3.90 mmol/L (4) poor image quality.

DM was diagnosed if a history of diabetes was reported in the medical record, if the patient had an HbA1c of ≥ 6.5% on admission, or if the patient was currently receiving antidiabetic medication [16]. According to the presence of concomitant DM, patients were further categorized into AMI (DM+) and AMI (DM−) groups. FBG was the first value obtained within 24 h of admission. Fasting SHR was calculated by dividing FBG by the estimated mean blood glucose level. Estimated mean plasma glucose level (mmo1/L) was calculated as follows: 1.59 × HbA1c (%) − 2.59 [8, 17]. According to the inclusion and exclusion criteria, a total of 357 patients with ASTEMI were included in this study. The patients were grouped as per their SHR tertiles and diabetes status. Data on clinical characteristics, medical history, serum biochemical parameters, angiography, and medication were collected from all patients.

### CMR protocol

All patients underwent CMR scanning with a 32-channel phased array coil under respiratory navigation and electrocardiographic gating. The scanning equipment was two 3.0 T CMR equipment (Achieva, Philips, Netherlands, Holland; Discovery MR750w, GE Healthcare, USA). Standardized imaging protocols included steady-state free precession breath-hold cine images, T2-weighted short-axis images, and late gadolinium enhancement (LGE).

The cine images were acquired via a steady-state free precession readout, with contiguous short-axis slices of both the left and right ventricles, extending from the mitral annulus to the apex. Long axis views (two-, three-, and four-chamber views) were also included, and each cardiac cycle comprised 25–30 phases. T2-weighted short-axis images were obtained using a short tau inversion recovery (STIR) sequence. Myocardial perfusion images were simultaneously acquired as 0.1 mmol/kg gadolinium chelate contrast agent was injected at a rate of 4 mL/s. Using a prospective ECG-gated gradient-echo sequence, short axis, two- and four-chamber LGE images were obtained 10–15 min after the intravenous injection of 0.2 mmol/kg gadolinium chelate contrast agent. The sequence parameters were as follows: repetition time/echo time: 4.1/1.6 ms; flip angle: 20°; image matrix: 256 × 130.

### CMR imaging analyses

Left ventricular function was analyzed using CVI42 commercial software (5.2.0, Circle, Canada). Cardiac function analysis was performed using the Cvi42 Short 3D module to semiautomatically identify and delineate epicardial and endocardial boundaries at end systole and diastole, including papillary muscles on cine short-axis sequences. Identified inaccuracies were modified and corrected by experts with > 10 years of experience in cardiovascular imaging diagnosis. The software automatically generated ventricular function parameters, such as left ventricular ejection fraction (LVEF), left ventricular end-diastolic volume (LVEDV), left ventricular end-systolic volume (LVESV), left ventricular stroke volume (LVSV), and left ventricular mass (LV-MASS). Left ventricular global function index (LVGFI) was defined according to the following formula for each subject: LVGFI = (LVSV/LVGV) × 100%. Left ventricular global volume (LVGV) was defined as the sum of the mean LV cavity volume (LVEDV + LVESV)/2 and the myocardial volume. The LV myocardial volume was calculated as the LV-MASS divided by the specific myocardial density (1.05 g/mL).

Left ventricular myocardial strain was analyzed using the Cvi42 Tissue Tracking module. End-diastolic cine images were selected, and the endocardium and epicardium of the delineated short-axis and long-axis cine images were automatically identified by the software. The inaccuracy was identified and corrected by experts, and left ventricular myocardial strain was automatically generated after the operation, including global radial (GRS), global circumferential (GCS), and global longitudinal (GLS) strains.

Regions with signal intensities of > 5 standard deviations above the normal myocardium on LGE short-axis images were defined as areas of LGE, and the mass of LGE (in grams) was automatically derived as the percentage of LV-MASS, i.e., infarction size. The T2w STIR infarct areas of low enhancement were identified as intramyocardial hemorrhage (IMH). MVO was defined as a low-intensity area within the high-enhancing myocardium. The extent of MVO was normalized to the percentage of total LV-MASS (% LV).

### Reproducibility analysis

Thirty patients were randomly selected to investigate intraobserver and interobserver agreement for left ventricular strain, including GRS, GCS, and GLS. Interobserver agreement: Ventricular strain was independently measured in 30 patients by a second radiologist experienced in CMR diagnosis who was blinded to the first observer’s results. Intraobserver agreement: Ventricular strain measurement was repeated in these 30 patients after an interval of 1 month by the same observer.

### Statistical analyses

Continuous variables were assessed for normality using the Shapiro–Wilk test. For continuous variables conforming to normal distribution, data were presented as mean ± standard deviation; data with a skewed distribution were given as median and interquartile range. Categorical variables were depicted as percentages and frequencies. For continuous variables conforming to normal distribution, differences in baseline and CMR characteristics among fasting SHR groups and diabetes status were compared using analysis of variance (ANOVA test) with equal variance. For continuous variables with unequal variance and those with non-normal distribution, Kruskal–Wallis test was used to compare the differences in baseline and CMR characteristics. Differences between groups were compared using the chi-square test for dichotomous variables. When there were significant differences, multiple comparisons were performed among the three stratified groups by SHR, corrected using Bonferroni method, and statistical differences were determined when the test level α′ = 0.05/3 = 0.0167 and *P* < 0.0167.

Spearman’s rho (r) was used to determine the relationship between fasting SHR and left ventricular function and myocardial strain in patients with AMI and in the subgroups AMI (DM−) and AMI (DM+). Three different multivariable linear regression analysis models [β coefficient (β)] were used to identify independent associations between fasting SHR or fasting SHR groups and left ventricular function and myocardial strain. In multivariable linear regression, confounders were prespecified based on clinical importance, previously published data, and statistical significance in univariable linear regression analysis.

Standardized fasting SHR using Z-score. Univariable logistic regression was used to analyze the impact of Z-score fasting SHR, clinical characteristics, and CMR parameters on the presence of MVO in patients with AMI and those with AMI (DM+). Clinical and CMR risk factors that were observed to be statistically significant in univariable logistic regression analysis (*P* < 0.05) were included in the multivariable logistic regression model. Correlations between covariates were evaluated using Spearman’s correlation coefficients. For variables with a correlation of > 0.7, one variable was selected for inclusion in the multivariable logistic regression model based on clinical experience and previous literature. Similarly, we analyzed the impact of fasting SHR groups, clinical data and CMR parameters on the presence of MVO in patients with AMI.

In addition, Spearman’s correlation coefficient was used to assess the relationship between fasting SHR and the extent of MVO. Univariable and multivariable linear regression models were used to determine the relationship between fasting SHR and the extent of MVO in patients with AMI and those with AMI (DM+).

Interobserver and intraobserver agreement for ventricular strain parameters were assessed using intraclass correlation coefficients (ICCs). ICC < 0.4 indicated poor agreement, and ICC > 0.75 signified good agreement.

*P* values < 0.05 were considered statistically significant (Statistical differences were determined at *P* < 0.0167 when performing multiple comparisons among the three stratified groups by SHR.). All the above statistical procedures were analyzed and plotted using IBM SPSS (version 25.0, IBM Corporation, Armonk, NY, USA).

**Table 2:** CMR characteristics of the study population

Variables	Overall (*n* = 357)	SHR < 0.85 (*n* = 121)	0.85 ≤ SHR < 1.01 (*n* = 115)	SHR ≥ 1.01 (*n* = 121)	*P* value
LVEF, %	48.7 ± 12.3	53.3 ± 12.3	48.0 ± 12.2^a^	45.0 ± 11.3^c^	**< 0.001**
LVGFI	28.0 ± 8.8	31.0 ± 9.4	27.8 ± 8.4^a^	25.3 ± 7.5^c^	**< 0.001**
LVEDV, ml	130.0 (103.0,156.4)	130.3 (95.0,162.1)	133.9 (111.0,158.5)	126.4 (102.3,150.4)	0.351
LVESV, ml	65.9 (47.6,87.4)	59.3 (40.4,90.7)	70.4 (47.9,88.0)	68.5 (50.9,85.2)	0.114
LVSV, ml	60.4 (48.2,74.4)	68.3 (52.8,78.4)	61.8 (49.0,76.7)	55.0 (43.3,65.7)^bc^	**< 0.001**
CO, l/min	4.4 (3.5,5.3)	4.7 (3.5,5.7)	4.7 (3.5,5.6)	4.1 (3.4,4.9)^bc^	**0.013**
LV-MASS, g	132.4 (111.2,155.4)	131.5 (103.9,153.6)	133.9 (111.7,155.5)	132.4 (115.7,157.2)	0.436
GRS, %	12.3 ± 3.4	13.5 ± 3.5	12.6 ± 2.9	11.1 ± 3.3^bc^	**< 0.001**
GCS, %	− 18.6 ± 6.5	− 20.2 ± 7.2	− 18.9 ± 5.7	− 16.5 ± 6.0^bc^	**< 0.001**
GLS, %	− 10.0 ± 3.4	− 10.5 ± 3.5	− 10.3 ± 3.0	− 8.9 ± 3.3^bc^	**< 0.001**
Infarct size (% LV mass)	28.0 (18.5,37.3)	24.7 (15.1,33.1)	29.9 (20.5,36.5)^a^	31.6 (21.7,42.4)^c^	**< 0.001**
Extent of MVO (% LV mass)	1.0 (0.0,3.5)	0.0 (0.0,2.4)	1.2 (0.0,3.3)^a^	2.1 (0.2,5.4)^bc^	**< 0.001**
Presence of MVO, n (%)	232 (65.0)	56 (46.3)	77 (67.0)^a^	99 (81.8)^bc^	**< 0.001**
Presence of IMH, n (%)	206 (57.7)	51 (42.1)	63 (58.4)	92 (76.0)^bc^	**< 0.001**
Location anterior, n (%)	158 (44.3)	49 (40.5)	52 (45.2)	57 (47.2)	0.568

*P* values < 0.05 indicate significance (bolded).

CMR: cardiac magnetic resonance; SHR: stress hyperglycemia ratio; LVEF: left ventricular ejection fraction; LVGFI: left ventricular global function index; LVEDV: left ventricular end diastolic volume; LVESV: left ventricular end systolic volume; LV: left ventricular; SV: stroke volume; CO: cardiac output; GRS: global radial strain; GCS: global circumferential strain; GLS: global longitudinal strain; MVO: microvascular obstruction; IMH: intramyocardial hemorrhage.

^a^*P* < 0.0167 for 0.85 ≤ SHR<1.01 vs. SHR < 0.85.

^b^*P* < 0.0167 for 0.85 ≤ SHR<1.01 vs. SHR ≥ 1.01.

^c^*P* < 0.0167 for SHR ≥ 1.01 vs. SHR < 0.85.

## Results

### Baseline characteristics

A total of 357 patients with ASTEMI were included in this study (Fig. 1). Of these, 132 patients were classified as DM and 225 as non-DM. Their median age was 56.9 ± 11.2 years, and 85.4% were men. The patients were grouped according to fasting SHR tertiles: SHR1: fasting SHR < 0.85 (*n* = 121); SHR2: 0.85 ≤ fasting SHR < 1.01 (*n* = 115); and SHR3: fasting SHR ≥ 1.01 (*n* = 121). The main clinical baseline characteristics of the study cohort are summarized in Table 1. The heart rate of patients in SHR3 was higher than that of those in SHR1 and SHR2. The number of previous/current smokers was higher in SHR2 than in SHR3. SHR3 had more patients with DM than the SHR2 group. Moreover, patients in SHR3 had the highest blood glucose level on admission. FBG increased with increasing fasting SHR tertiles. HbA1c was higher in SHR1 than in SHR2. In addition, creatine kinase-myocardial band (CKMB) masses of SHR2 and SHR3 were higher than those of SHR1. Nonetheless, no significant differences were observed among the three groups in terms of body mass index, blood pressure, other clinical and biochemical parameters, and surgical and discharge medications. Supplementary Table 1 showed baseline characteristics by diabetes status. ABG, FBG, and HbA1c levels were higher in patients with AMI (DM+) than in patients with AMI (DM−).

**Fig. 2:**
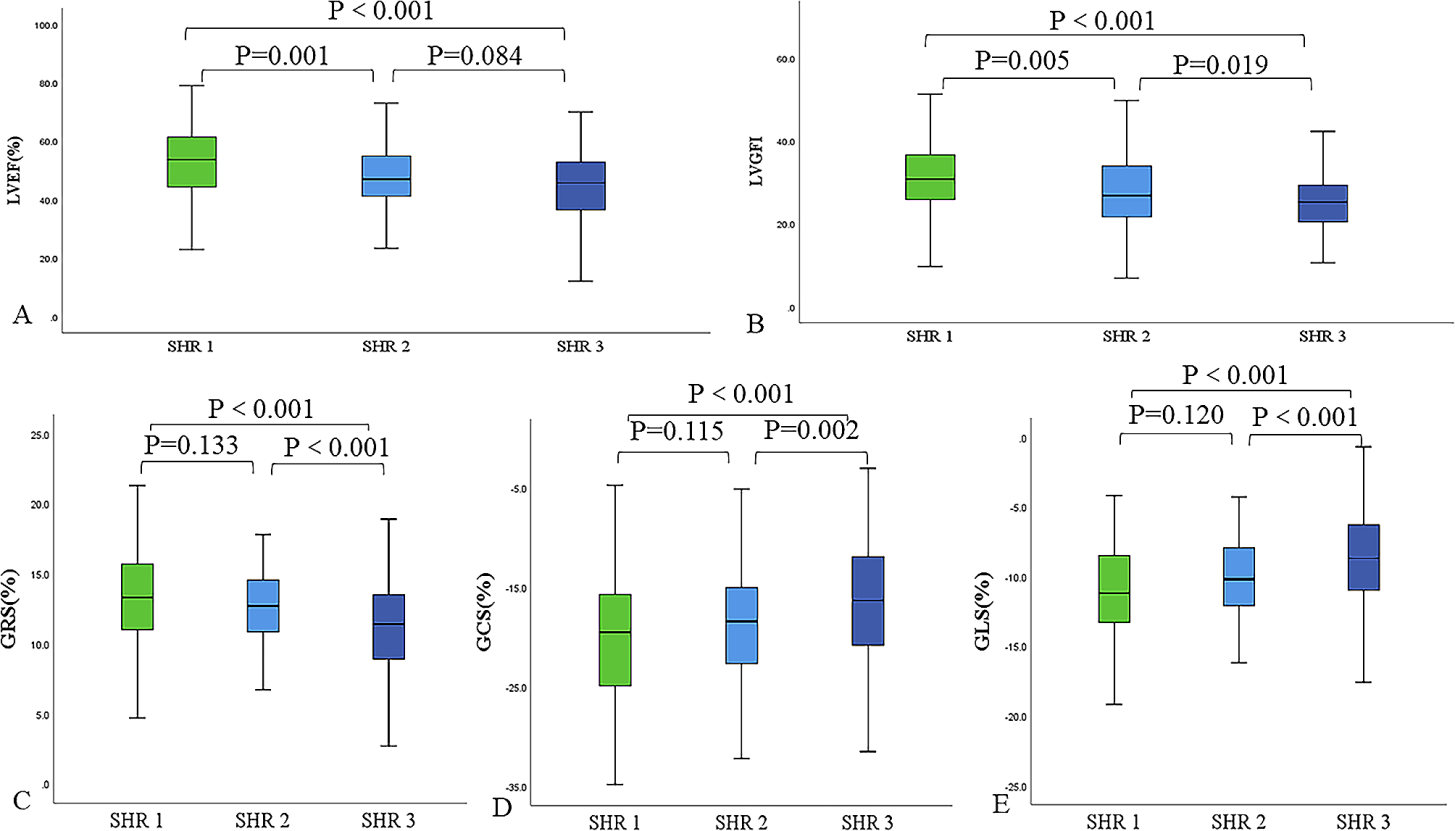
Box plot for the comparison of LVEF, LVGFI, and myocardial strain among the three groups as determined using fasting SHR. *P* values represent differences between groups. SHR1: fasting SHR<0.85; SHR2: 0.85 ≤ fasting SHR<1.01; SHR3: fasting SHR ≥ 1.01. SHR: stress hyperglycemia ratio; LVEF: left ventricular ejection fraction; LVGFI: left ventricular global function index; GRS: global radial strain; GCS: global circumferential strain; GLS: global longitudinal strain. *P* values < 0.0167 were considered statistically significant

**Fig. 3:**
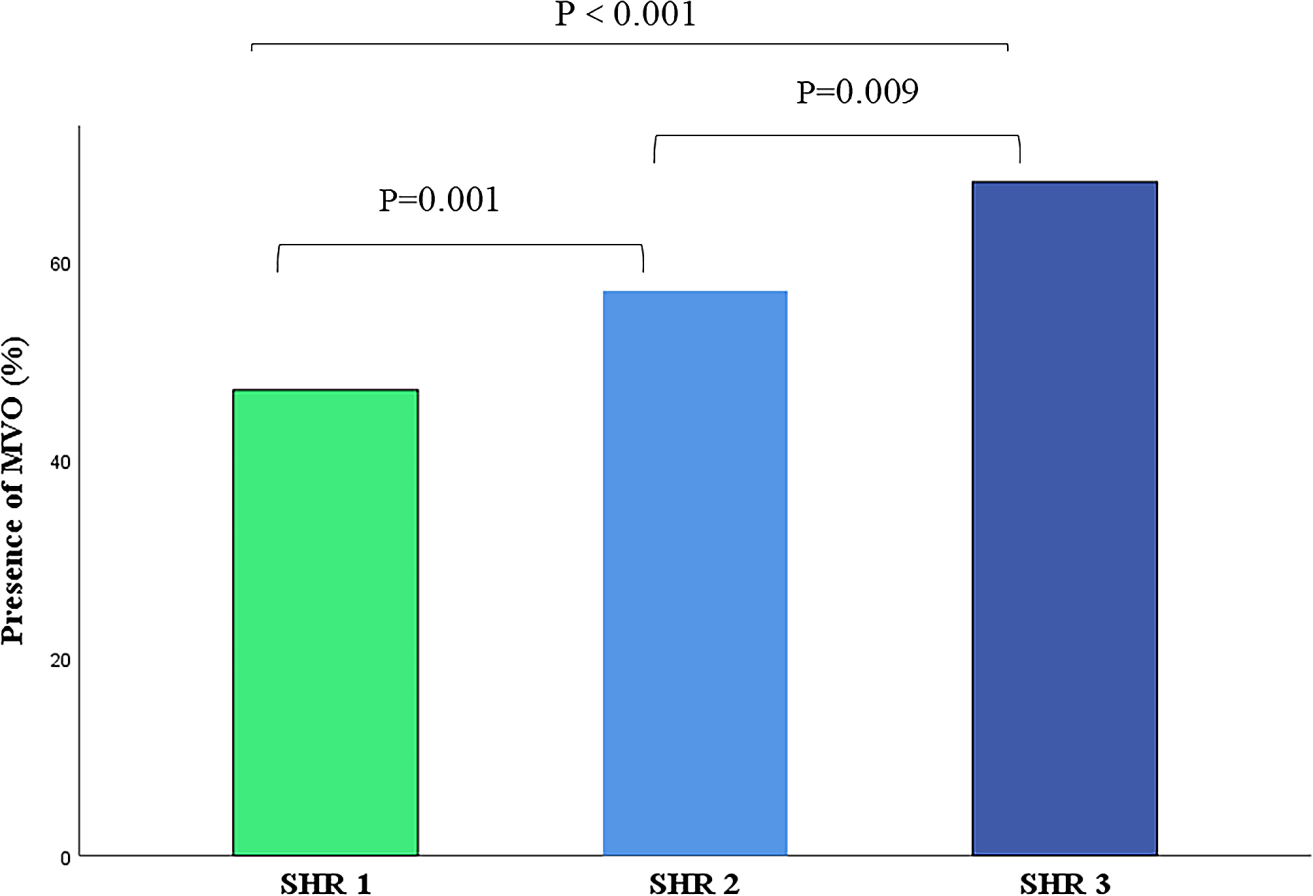
Bar chart of the comparison of MVO presence among the three groups as determined using fasting SHR. *P* values represent differences between groups. SHR1: fasting SHR<0.85; SHR2: 0.85 ≤ fasting SHR<1.01; SHR3: fasting SHR ≥ 1.01. *P* values < 0.0167 were considered statistically significant. SHR: stress hyperglycemia ratio; MVO: microvascular obstruction

**Fig. 4:**
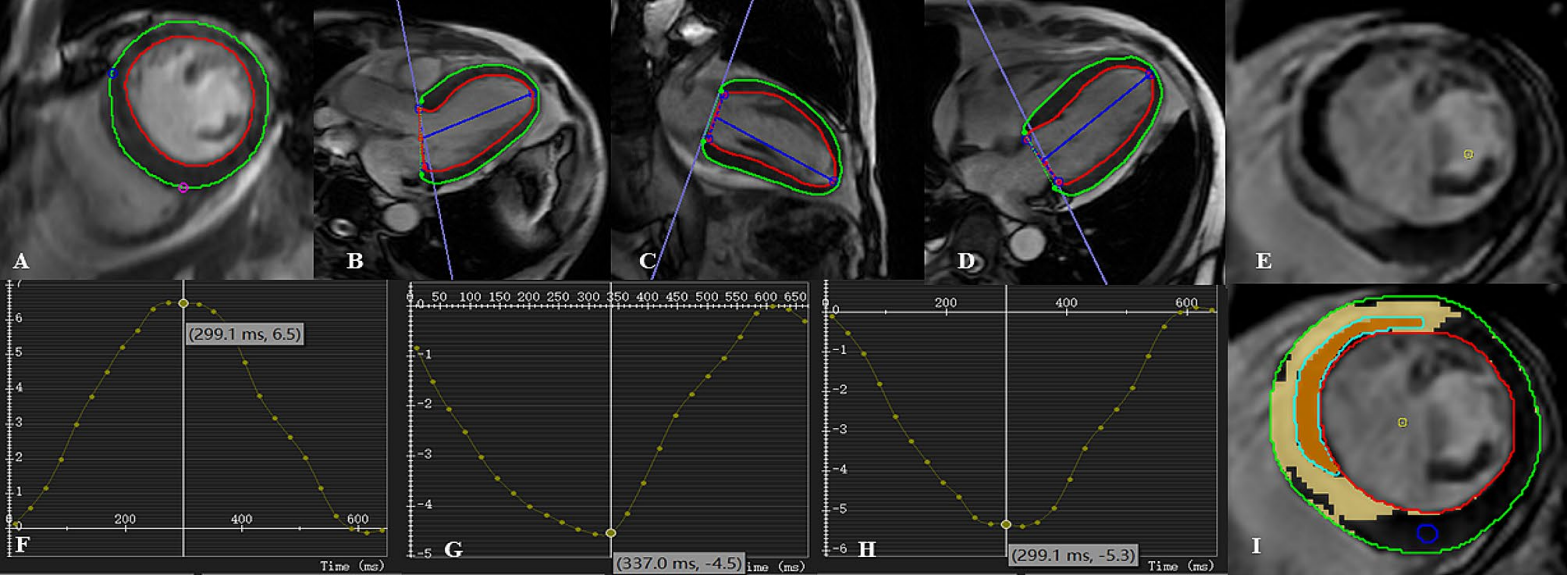
The assessment included measurements of left ventricular myocardial strain, the extent of MVO, and the infarct size in the representative case. SHR: stress hyperglycemia ratio; GRS: global radial strain; GCS: global circumferential strain; GLS: global longitudinal strain; MVO: microvascular obstruction

### Comparison of CMR findings among the groups (SHR1, SHR2, and SHR3)

Table 2 summarizes the CMR characteristics of patients grouped by fasting SHR tertiles and for all patients. LVEF and LVGEI was significantly lower in SHR2 (48.0% ± 12.2%; 27.8 ± 8.4) and SHR3 (45.0% ± 11.3%; 25.3 ± 7.5) than in SHR1 (53.3% ± 12.3%; 31.0 ± 9.4). The absolute values of SV, CO, GRS, GCS, and GLS were the lowest in SHR3 (Fig. 2). The infarct size was higher in SHR2 and SHR3 than in SHR1. The extent of MVO increased as the group increased. Furthermore, the number of patients with MVO increased with increasing group (Fig. 3). More patients had IMH in SHR3 than in SHR1 and SHR2. However, there were no significant differences in EDV, ESV, and LV-MASS among the three groups. Figure 4 shows a representative case: male, fasting SHR = 1.26, SHR3. Strain analysis was performed on a series of short-axis images, along with two-, three-, and four-chamber views at end diastole (A–D). F–G depict the results of GRS, GCS, and GLS. E and H illustrate the MVO% and LGE% measurement methods, and their values were 16.61% and 30.44%, respectively. Supplementary Table 2 showed CMR characteristics by diabetes status, which revealed that compared to patients with AMI (DM−), patients with AMI (DM+) exhibited significantly lower absolute values in LVEF, LVGFI, LVSV, GRS, GLS and GCS (*P* < 0.05).

### Association between fasting SHR and left ventricular function and strain

Fasting SHR was negatively correlated with LVEF, LVGFI, and GRS (*r* = − 0.252; *r* = − 0.261; and *r* = − 0.245) and positively correlated with GCS (*r* = 0.221) and GLS (*r* = 0.249) (all *P* < 0.001). After adjusting for the covariates of age, sex, heart rate, diabetes, Killip class, location of culprit lesion and TIMI flow grade 0 or 1 pre-percutaneous coronary intervention (PCI), oral hypoglycemic drugs, insulin therapy, HbA1C%, CKMB mass, myoglobin, brain natriuretic peptide (BNP), high-sensitive C-reactive-protein (hsCRP), LVMASS, infarct size, and extent of MVO, fasting SHR was found to be an independent determinant of impaired LVEF, LVGFI, GRS, and GLS (Table 3), with β = − 6.815, − 5.403, − 1.330, and 1.375, respectively. And after correcting for other confounders, LVEF decreased by 3.915%, LVGFI decreased by 2.971, GRS decreased by 0.753%, and GLS increased by 0.862% in patients with SHR3 compared patients with SHR1 (Supplementary Table 3).

**Table 3:** Associations of fasting SHR with left ventricular function and strain

	LVEF			LVGFI			GRS			GCS			GLS		
	Beta Coefficient	Standard Error	*P* Value	Beta Coefficient	Standard Error	*P* Value	Beta Coefficient	Standard Error	*P* Value	Beta Coefficient	Standard Error	*P* Value	Beta Coefficient	Standard Error	*P* Value
Crude	− 15.801	2.992	< 0.001	− 10.843	2.136	< 0.001	− 3.994	0.825	< 0.001	6.986	1.559	< 0.001	3.637	0.825	< 0.001
Model 1	11.718	2.870	< 0.001	− 7.973	1.994	< 0.001	− 2.491	0.744	0.001	4.073	1.425	0.005	2.053	0.721	0.005
Model 2	− 8.945	2.636	0.001	− 6.343	1.886	0.001	− 1.828	0.687	0.008	2.717	1.303	0.038	1.696	0.691	0.015
Model 3	− 6.815	2.591	0.009	− 5.403	1.810	0.003	− 1.330	0.646	0.040	2.001	1.250	0.110	1.375	0.673	0.042

Model 1: adjusted for Age, Sex, Heart rate, Diabetes, Killip class, Location of culprit lesion and TIMI flow grade 0/1 pre-PCI, Oral hypoglycemic drugs, Use insulin. Model 2: adjusted for model 1 covariates + HbA1c, CKMBmass, Myoglobin, BNP and High-sensitive CRP. Model 3: adjusted for model 2 covariates + LVMASS, Infarct size and extent of MVO.SHR: stress hyperglycemia ratio; LVEF: left ventricular ejection fraction; LVGFI: left ventricular global function index; GRS: global radial strain; GCS: global circumferential strain; GLS: global longitudinal strain; TIMI: thrombolysis in myocardial infarction; HbA1c: glycated hemoglobin A1c; CKMB: creatine kinase-myocardial band; BNP: brain natriuretic peptide; CRP: C-reactive-protein; MVO: microvascular obstruction. *P* values < 0.05 indicate significance.

**Table 4:** Univariable and multivariable logistic regression analysis of fasting SHR and presence of MVO in AMI and AMI(DM+)

	Univariable analysis in AMI		Multivariable analysis in AMI		Univariable analysis in AMI(DM+)		Multivariable analysis in AMI (DM+)	
	OR (95% CI)	*P* value	OR (95% CI)	*P* value	OR (95% CI)	*P* value	OR (95% CI)	*P* value
Male, n (%)	2.281 (1.259,4.135)	0.007	…	…	…	…	…	…
Heart rate, bpm	1.025 (1.007,1.043)	0.007	…	…	…	…	…	…
Z-Score Fasting SHR	1.980 (1.499,2.617)	< 0.001	1.591 (1.105,2.289)	0.012	1.870 (1.197,2.921)	0.006	2.557 (1.239,5.274)	0.011
CKMBmass, ng/ml	1.009 (1.006,1.011)	< 0.001	1.006 (1.002,1.009)	0.001	1.011 (1.006,1.015)	0.000	1.010 (1.003,1.017)	0.005
Myoglobin, ug/L	1.001 (1.000,1.002)	0.019	0.999 (0.998,1.000)	0.042	…	…	…	…
BNP, pg/ml	1.002 (1.001,1.003)	0.003	…	…	…	…	…	…
High-sensitive CRP, mg/L	1.083 (1.043,1.125)	< 0.001	1.065 (1.014,1.119)	0.012	1.088 (1.017,1.163)	0.014	…	…
TIMI flow grade 0/1 pre-PCI, n (%)	0.248 (0.148,0.417)	< 0.001	0.429 (0.213,0.863)	0.018	0.252 (0.112,0.565)	0.001	…	…
oral hypoglycemic drugs, n (%)	0.508 (0.293,0.879)	0.016	…	…	…	…	…	…
LVEF, %	0.927 (0.907,0.948)	< 0.001	0.954 (0.926,0.983)	0.002	0.935 (0.901,0.970)	0.000	…	…
LV-MASS, g	1.012 (1.005,1.019)	< 0.001	…	…	1.014 (1.003,1.026)	0.015	…	…
Infarct size (% LV mass)	1.115 (1.087,1.144)	< 0.001	1.082 (1.050,1.115)	<0.001	1.144 (1.089,1.202)	0.000	1.095 (1.035,1.159)	0.002
Location anterior, n (%)	0.624 (0.400,0.974)	0.038	…	…	…	…	…	…

OR: odds ratio; CI: confidence interval; SHR: stress hyperglycemia ratio; AMI: acute myocardial infarction; DM: Diabetes; CKMB: creatine kinase-myocardial band; BNP: brain natriuretic peptide; CRP: C-reactive-protein; TIMI: thrombolysis in myocardial infarction; PCI: percutaneous coronary intervention; LVEF: left ventricular ejection fraction. *P* values < 0.05 indicate significance

### Association between fasting SHR and MVO

Univariable logistic regression showed that sex, heart rate, Z-score fasting SHR/fasting SHR groups, CKMB mass, myoglobin, BNP, hsCRP, pre-PCI TIMI grade 0 or 1, oral hypoglycemic drugs, LVEF, LV-MASS, infarct size and location anterior were statistically significant in predicting the presence of MVO in patients with AMI. After multivariable logistic regression adjusted for the above parameters, Z-score fasting SHR remained an independent predictor of the presence of MVO (*P* = 0.012, OR = 1.591 (1.105,2.289) (Table 4). After adjusting for other confounders, the risk of MVO presence was 3.878-fold higher in patients with SHR3 than patients with SHR1, with a 95% CI of 1.766, 8.514, *P* = 0.001 (Supplementary Table 4). Spearman’s rho testing revealed that the extent of MVO and fasting SHR were associated; *r* = 0.269, *P* < 0.001. Furthermore, multivariable linear regression showed that fasting SHR was independently associated with the extent of MVO after adjusting for confounding factors (Table 5).

**Fig. 5:**
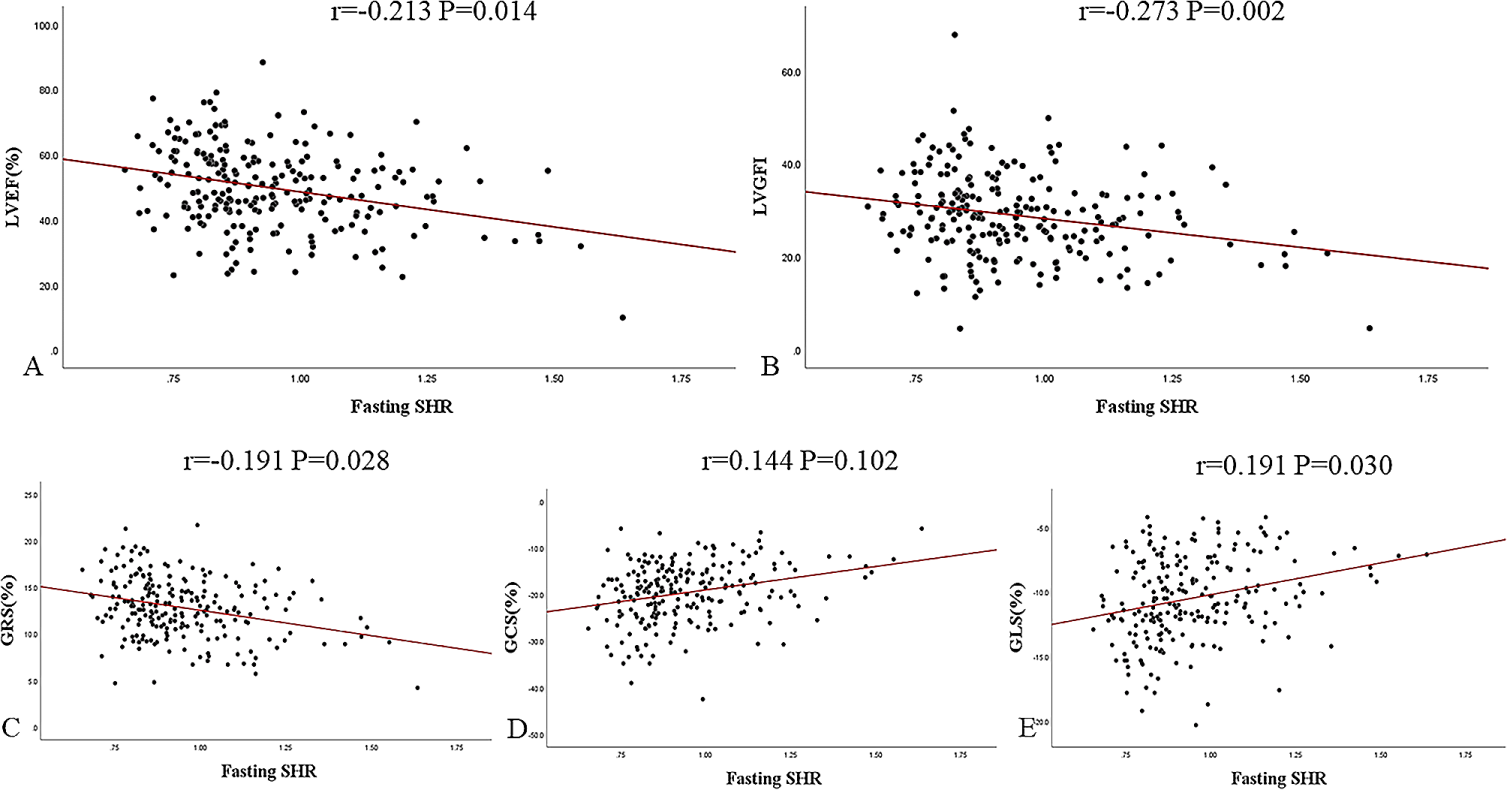
Scatter plots depicting the association of fasting SHR with LVEF, LVGFI, and myocardial strain in AMI (DM+). The Spearman’s correlation coefficient r and corresponding *P* values are shown. AMI: acute myocardial infarction; DM: diabetes mellitus; SHR: stress hyperglycemia ratio; LVEF: left ventricular ejection fraction; LVGFI: left ventricular global function index; GRS: global radial strain; GCS: global circumferential strain; GLS: global longitudinal strain

**Fig. 6:**
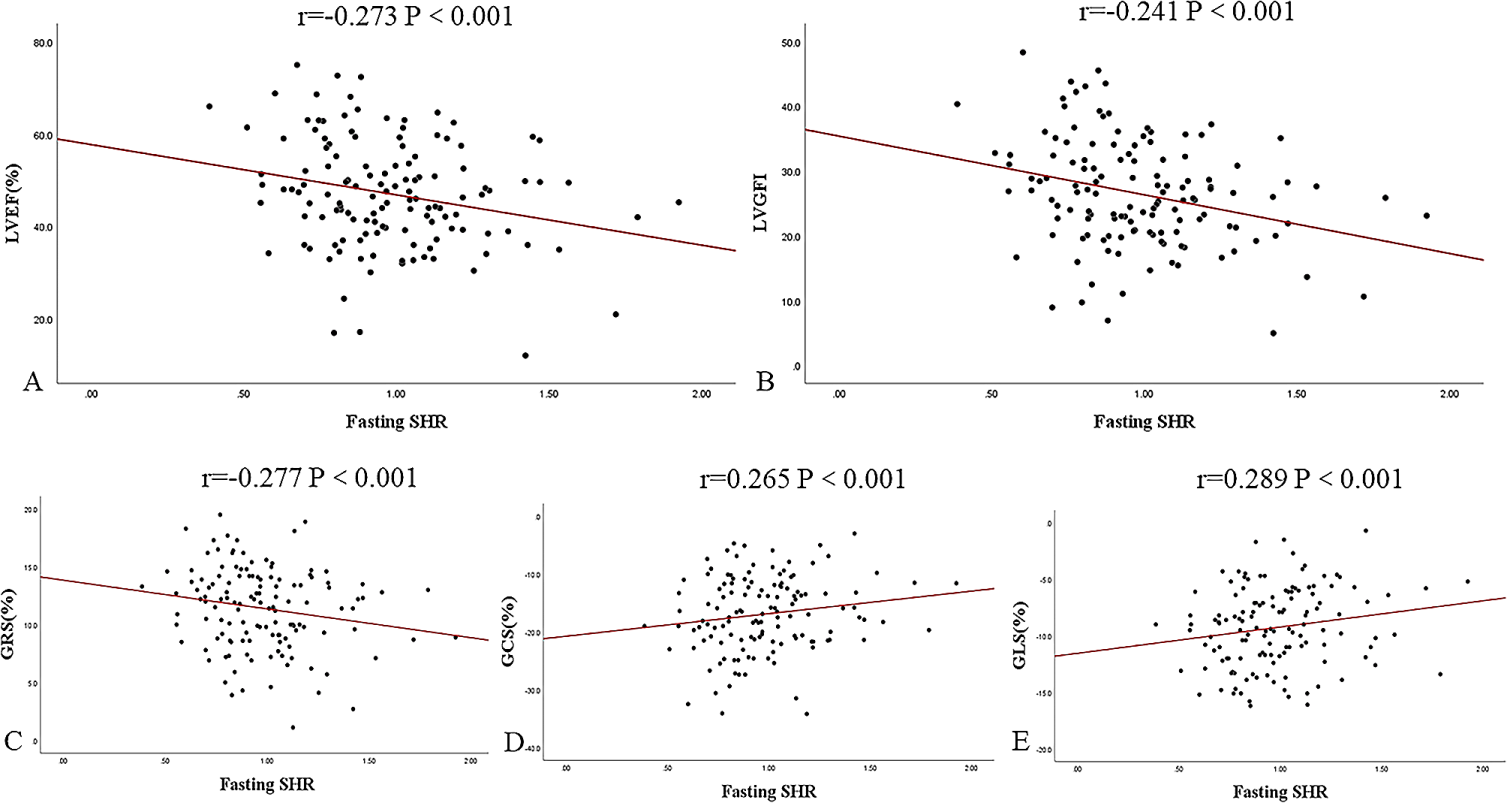
Scatter plots depicting the association of fasting SHR with LVEF, LVGFI, and myocardial strain in AMI (DM−). The Spearman’s correlation coefficient r and corresponding *P* values are shown. AMI: acute myocardial infarction; DM: diabetes mellitus; SHR: stress hyperglycemia ratio; LVEF: left ventricular ejection fraction; LVGFI: left ventricular global function index; GRS: global radial strain; GCS: global circumferential strain; GLS: global longitudinal strain

**Table 5:** Univariable and multivariable linear regression analysis between fasting SHR and extent of MVO in AMI and AMI(DM+)

Variables	Univariable analysis in AMI			Multivariable analysis in AMI			Univariable analysis in AMI (DM+)			Multivariable analysis in AMI (DM+)		
	Beta coefficient	Standard error	*P* Value	Beta coefficient	Standard error	*P* Value	Beta coefficient	Standard error	*P* Value	Beta coefficient	Standard error	*P* Value
Fasting SHR	5.328	1.141	< 0.001	3.008	1.092	0.006	5.879	1.848	<0.001	4.288	1.848	0.024
CKMB mass, ng/ml	0.007	0.002	< 0.001	…	…	…	0.017	0.005	<0.001	…	…	…
Myoglobin, ug/L	0.002	0.001	0.004	…	…	…	0.002	0.001	0.002	…	…	…
BNP, pg/ml	0.004	0.001	0.002	…	…	…	…	…	…	…	…	…
LDL cholesterol, mmol/L	0.611	0.256	0.017	0.487	0.235	0.039	…	…	…	…	…	…
Number of diseased arteries,	− 0.596	0.298	0.046	…	…	…	…	…	…	…	…	…
Culprit LAD	Reference						Reference					
Culprit LCX	− 0.946	0.774	0.223	…	…	…	− 2.524	1.653	0.129	…	…	…
Culprit RCA	− 1.655	0.543	0.002	…	…	…	− 2.424	1.063	0.024	…	…	…
TIMI flow grade 0/1 pre-PCI	2.357	0.578	< 0.001	1.126	0.553	0.042	2.842	0.011	<0.001	…	…	…
Oral hypoglycemic drugs	2.494	0.576	< 0.001	…	…	…	…	…	…	…	…	…
Insulin	2.761	0.752	< 0.001	…	…	…	…	…	…	…	…	…
LVEF, %	− 0.118	0.019	< 0.001	…	…	…	− 0.128	0.041	<0.001	…	…	…
LV-MASS, g	0.020	0.007	0.005	…	…	…	…	…	…	…	…	…
Infarct size (% LV mass)	0.144	0.016	< 0.001	0.092	0.020	<0.001	0.156	0.031	<0.001	0.078	0.039	0.048

SHR: stress hyperglycemia ratio; AMI: acute myocardial infarction; DM: Diabetes; CKMB: creatine kinase-myocardial band; BNP: brain natriuretic peptide; LDL: low-density lipoprotein; LAD: left anterior descending; LCX: left circumflex artery; RCA: right coronary artery; TIMI: thrombolysis in myocardial infarction; PCI: percutaneous coronary intervention; LVEF: left ventricular ejection fraction. *P* values < 0.05 indicate significance

### Subgroup analysis

In the AMI (DM+) group, fasting SHR was negatively correlated with LVEF, LVGFI, and GRS (r = − 0.213, *P* = 0.014; r = − 0.273, *P* = 0.002; and r = − 0.191, *P* = 0.028) and positively correlated with GLS (r = 0.191, *P* = 0.028) (Fig. 5). In the AMI (DM−) group, fasting SHR was correlated with LVEF, LVGFI, GRS, GCS, and GLS by − 0.273, − 0.241, − 0.277, 0.265, and 0.289, all *P* values < 0.001 (Fig. 6).

In AMI (DM+), multivariable logistic regression showed that Z-score fasting SHR was independently associated with the presence of MVO after adjusting for confounding factors (*P* = 0.011) (Table 4). Spearman’s rho testing indicated that the extent of MVO and fasting SHR were associated with each other, *r* = 0.292, *P*<0.001. Fasting SHR was independently and positively linked to the extent of MVO after correction for CKMB mass, myoglobin, location of the culprit lesion, TIMI flow grade 0 or 1 pre-PCI, LVEF, and infarct size, *P* = 0.024 (Table 5).

### Intraobserver and interobserver variability

Left ventricular strain had good intraobserver and interobserver repeatability. The interobserver ICCs of GRS, GCS, and GLS were 0.978, 0.986, and 0.970 respectively, and the intraobserver ICCs were 0.961, 0.970, and 0.964 respectively, with *P* values < 0.001.

## Discussion

This study investigated the combined effects of fasting SHR on left ventricular function, strain, and MVO in patients with AMI. The key findings were as follows: (1) Patients with high fasting SHR exhibited significantly impaired left ventricular function and strain, and fasting SHR was found to be an independent determinant of impaired LVEF, LVGFI, and global peak strain in radial and longitudinal directions. In addition, in both AMI (DM+) and AMI (DM−) subgroups, fasting SHR was associated with impaired left ventricular function and strain. (2) Fasting SHR demonstrated independent predictive value for the presence of MVO, and it was correlated with the extent of MVO. (3) In the AMI (DM+) group, fasting SHR was independently correlated with both the presence and extent of MVO. These results may provide a potential pathophysiological mechanism for the relationship between fasting SHR and poor prognosis after ASTEMI, especially in the AMI (DM+) group.

During AMI, the elevation of glucagon, cortisol, and cytokines encourages glucose production via increased gluconeogenesis and glycogenolysis. Nevertheless, the inadequate insulin secretion from pancreatic β-cells cannot counteract the hyperglycemic effects of these counter-regulatory hormones and cytokines, ultimately resulting in stress-induced hyperglycemia [18, 19]. SH has been reported to be a powerful predictor of increased mortality and morbidity risk in patients with AMI [8, 20, 21]. Introduced as a novel marker of relative hyperglycemia, SHR has a better ability to discern adverse outcomes than ABG alone in AMI because it controls background glucose levels [1, 22]. Fasting SHR avoids the effect of meal timing and appears to play a pertinent role in prognosis assessment [12]. Currently, there are no studies on the association of fasting SHR with left ventricular function and MVO.

Acute glucose excursions lead to increased oxidative stress, causing endothelial dysfunction, vascular inflammation, and activation of coagulation, thereby worsening myocardial injury [23, 24]. This study observed that fasting SHR was independently associated with both LVGFI and LVEF. LVGFI assessment combines factors associated with left ventricular chamber size and mass and reflects cardiac remodeling under stress in patients with AMI, suggesting the effect of SH on myocardial injury [25, 26]. Several studies have asserted that myocardial strain in the acute phase after ASTEMI sensitively predicts adverse left ventricular remodeling and clinical outcomes [27, 28]. In this study, fasting SHR was independently associated with impaired global peak radial and longitudinal strains, which implies that high fasting SHR causes corresponding myocardial injury and that this effect is mainly concentrated in subendocardial myocardial fibers. In addition, our results showed that patients in SHR3 group had more serious deterioration of cardiac function, GRS and GLS than those in SHR1 group, which is consistent with previous studies that patients in the higher SHR group tend to have a worse prognosis [9]. It suggests that more attention should be paid to patients with high SHR group in clinical practice.

MVO was independently associated with left ventricular remodeling and poor prognosis in patients with AMI [29]. MVO was initially described as altered myocardial blush grade in invasive coronary angiography. Severe MVO may significantly reduce the flow in patent upstream epicardial arteries, which is called the no-reflow phenomenon. Iwakura et al. [30] investigated the effects of hyperglycemia on admission and no-reflow phenomenon and found that hyperglycemia was the strongest predictor of no reflow. CMR was excellently correlated with histological reference and can noninvasively and quantitatively assess MVO. Cochet [31] evaluated the CMR features in 113 patients with ASTMI treated successfully using PCI and found that hyperglycemia at admission was independently associated with the extent of MVO, as assessed using CMR. This study suggested that SH was closely related to MVO. After adjusting for other confounders, fasting SHR was an independent predictor of the presence of MVO. In addition, it was an independent determinant of the extent of MVO. The possible mechanisms are as follows: Acute hyperglycemia also increases the levels of intercellular adhesion molecule-1 and P-selectin, which in turn enhance the lodging of leukocytes in the capillaries. The increased leukocyte lodging in the microcirculation might exacerbate the no-reflow phenomenon [32, 33]. Moreover, hyperglycemia may reduce the protective effect of ischemic preconditioning by impeding mitochondrial ATP-regulated K channel activation [34]. This study alluded that MVO is a potential mechanism between fasting SHR and myocardial injury.

Several studies [35, 36] have shown that SH is associated with larger infarcts, more pronounced reperfusion injury, and left ventricular dysfunction in patients with AMI (DM−). Eitel et al. [36] highlighted that hyperglycemia in patients with ASTEMI and previously undiagnosed diabetes was a stronger indicator of myocardial injury assessed using CMR than established diabetes. This observation could be attributed to the fact that in the above study, SH was assessed based on hyperglycemia at admission. In patients with diabetes, blood glucose level at admission is affected by chronic blood glucose level and eating and does not reflect the stress status adequately in those with ASTEMI. Recently, Cui’s findings [12] from the China Acute Myocardial Infarction Registry showed that high fasting SHR was significantly associated with higher in-hospital mortality in patients with AMI with or without diabetes. Our finding that fasting SHR is independently associated with the presence and extent of MVO confirmed the additive effect of stress-induced hyperglycemia on myocardial injury in patients with DM. This result suggests that the pathophysiological mechanism of high fasting SHR could be responsible for the poor prognosis of patients with AMI (DM+). This observation may improve the risk prediction using conventional risk factor models in this population.

Overall, this study observed an association between fasting SHR and myocardial injury (left ventricular function, myocardial strain, and MVO), which emphasized the importance of glycemic control and the necessity of giving more attention to patients with high SHR among those diagnosed with AMI. Furthermore, optimal thresholds for glycemic control and the possible beneficial effects of aggressive glycemic control on myocardial injury and prognosis should be investigated in the future in patients with AMI.

### Limitations

First, this was a retrospective single-center study; although the existing confounders were adjusted, selection bias and other potential confounders might have influenced the results. Certain baseline data, such as the duration of diabetes and treatment history, were not available. In addition, this study failed to distinguish between patients with prediabetes and non-diabetes; hence, patients with prediabetes could not be analyzed as a separate subgroup. Finally, the prognostic impact of fasting SHR and CMR parameters was not a goal of this study. Further studies are therefore required to investigate the prognostic impact of the combination of fasting SHR and CMR parameters in patients with AMI in a larger study population.

## Conclusions

Fasting SHR was independently associated with impaired left ventricular function and myocardial strain in patients with ASTEMI. Moreover, fasting SHR was an independent determinant of the presence and extent of MVO in patients with AMI and AMI (DM+). The above conclusions revealed a possible mechanism of action between fasting SHR and poor prognosis, thus implying that therapies targeting fasting SHR may be beneficial in these patients.

### Electronic supplementary material

Below is the link to the electronic supplementary material.

Supplementary Material 1.

Supplementary Material 2.

Supplementary Material 3.

Supplementary Material 4.

## Data Availability

No datasets were generated or analysed during the current study.

## Abbreviations

SH: Stress hyperglycemia
AMI: Acute myocardial infarction
MVO: Microvascular obstruction
ABG: Admission blood glucose
DM: Diabetes mellitus
SHR: Stress hyperglycemia ratio
HbA1c: Glycated hemoglobin A1c
CMR: Cardiac magnetic resonance
ASTEMI: Acute ST-segment elevation myocardial infarction
PPCI: Primary percutaneous coronary intervention
LGE: Late gadolinium enhancement
LVEF: Left ventricular ejection fraction
LVEDV: Left ventricular end-diastolic volume
LVESV: Left ventricular end-systolic volume
LVSV: Left ventricular stroke volume
LVMASS: Left ventricular mass
LVGFI: Left ventricular global function index
LVGV: Left ventricular global volume
GRS: Global radial strain
GCS: Global circumferential strain
GLS: Global longitudinal strain
IMH: Intramyocardial hemorrhage
ICC: Intraclass correlation coefficient
CKMB: Creatine kinase-myocardial band
hsCRP: High-sensitive C-reactive-protein
