# Supplementary material for: Association of stress hyperglycemia ratio with left ventricular function and microvascular obstruction in patients with ST-segment elevation myocardial infarction: a 3.0 T cardiac magnetic resonance study
Source: Cardiovasc Diabetol. 2024 May 27;23:179. doi: 10.1186/s12933-024-02271-6 (PMC11131267; doi:10.1186/s12933-024-02271-6)
Supplement: Supplementary file 1 — Supplementary Material 1. [file 12933_2024_2271_MOESM1_ESM.docx]

Baseline Characteristics of the Study Population by Diabetes Status

| Variables | Overall  (n=357) | Diabetes  (n=132) | | No Diabetes  (n=225) | P value |
| --- | --- | --- | --- | --- | --- |
| Age, years | 56.9±11.2 | | 58.1±10.5 | 56.1±11.5 | 0.062 |
| Male, n (%) | 305 (85.4) | | 107 (81.1) | 198 (88.0) | 0.073 |
| BMI, kg/m2 | 26.0±3.3 | | 25.9±3.6 | 26.0±3.2 | 0.373 |
| Systolic blood pressure, mmHg | 122.3±17.8 | | 124.9±17.1 | 120.8±18.0 | 0.046 |
| Diastolic blood pressure, mmHg | 76.0±11.6 | | 76.5±10.8 | 75.8±12.0 | 0.392 |
| Heart rate, bpm | 78.3±12.9 | | 79.3±12.9 | 77.8±12.9 | 0.191 |
| Previous/current smoker, n (%) | 233 (65.3) | | 76 (57.6) | 157 (69.8) | **0.019** |
| Current smoker, n (%) | 208 (58.3) | | 73 (55.3) | 135 (60.0) | **0.020** |
| Hypertension, n (%) | 219 (61.3) | | 93 (70.5) | 126 (56.0) | **0.007** |
| Dyslipidemia, n (%) | 243 (68.1) | | 85 (64.4) | 158 (70.3) | 0.254 |
| Prior myocardial infarction, n (%) | 11 (3.1) | | 3 (2.3) | 8 (3.6) | 0.498 |
| Previous PCI or CABG, n (%) | 19 (5.3) | | 8 (6.1) | 11 (4.9) | 0.634 |
| Killip class, n (%) |  | |  |  | 0.621 |
| I | 263 (73.7) | | 94 (71.2) | 169 (75.1) |  |
| II | 83 (23.2) | | 32 (24.2) | 51 (22.7) |  |
| III | 4 (1.1) | | 2 (1.5) | 2 (0.9) |  |
| IV | 7 (2.0) | | 4 (3.0) | 3 (1.3) |  |
| Blood results |  | |  |  |  |
| Blood glucose on admission mmol/L | 8.3 (6.9,11.5) | | 12.7 (9.7,15.6) | 7.3 (6.4,8.5) | **＜0.001** |
| Fasting blood glucose, mmol/L | 6.4 (5.5,8.8) | | 9.6 (7.3,13.2) | 5.9 (5.3,6.8) | **＜0.001** |
| HbA1c, % | 6.0 (5.6,7.2)) | | 7.9 (6.9,9.3) | 5.7 (5.5,6.0) | **＜0.001** |
| Fasting SHR | 0.9 (0.8,1.1) | | 1.0 (0.8,1.1) | 0.9 (0.8,1.0) | 0.306 |
| CKMB mass, ng/ml | 196.5 (86.0,303.0) | | 159.8 (62.1,259.0) | 215.3 (101.4,303.0) | **0.001** |
| Myoglobin, ug/L | 221.6 (64.0,473.8) | | 155.0 (53.0,452.3) | 256.3 (73.0,490.0) | 0.055 |
| BNP, pg/ml | 178.0 (81.5,316.0) | | 178.4 (87.5,305.3) | 178.0 (80.0,317.0) | 0.828 |
| Creatinine, umol/L | 72.0 (63.8,84.0) | | 71.6 (63.0,81.1) | 72.2 (64.1,84.9) | 0.418 |
| eGFR, mL/min/1.73 m2 | 97.7 (87.9,107.3) | | 96.5 (86.2,105.9) | 99.0 (88.4,108.3) | 0.129 |
| Triglycerides, mmol/L | 1.5 (1.1,2.1) | | 1.5 (1.1,2.1) | 1.4 (1.1,2.0) | 0.369 |
| Total cholesterol, mmol/L | 4.6 (4.0,5.5) | | 4.7 (4.2,5.6) | 4.6 (4.0,5.4) | 0.363 |
| HDL cholesterol, mmol/L | 1.0 (0.9,1.2)) | | 1.0 (0.9,1.2) | 1.0 (0.9,1.2) | 0.604 |
| LDL cholesterol, mmol/L | 3.0 (2.4,3.6) | | 3.1 (2.5,3.6) | 2.9 (2.4,3.6) | 0.681 |
| High-sensitive CRP, mg/L | 5.1 (2.3,11.2) | | 5.4 (2.3,11.2) | 5.0 (2.3,11.2) | 0.714 |
| Procedures |  | |  |  |  |
| Number of diseased arteries, n (%) 0.639 | | | | | |
| 1 | 145 (40.6) | | 50 (37.9) | 95 (42.2) |  |
| 2 | 108 (30.3) | | 40 (30.3) | 68 (30.2) |  |
| 3 | 104 (29.1) | | 42 (31.8) | 62 (27.6) |  |
| Location of culprit lesion, n (%) 0.750 | | | | | |
| LAD | 189 (52.9) | | 71 (53.8) | 118 (52.4) |  |
| LCX | 44 (12.3) | | 14 (10.6) | 30 (13.3) |  |
| RCA | 124 (34.7) | | 47 (35.6) | 77 (34.2) |  |
| TIMI flow grade 0/1 pre-PCI, n (%) | 276 (77.3) | | 96 (72.7) | 88 (76.5) | 0.113 |
| TIMI flow grade 3 post-PCI, n (%) | 344 (96.4) | | 129 (97.7) | 215 (95.6) | 0.290 |
| Medications |  | |  |  |  |
| Aspirin, n (%) | 338 (94.7) | | 122 (92.4) | 216 (96.0) | 0.146 |
| P2Y12receptor inhibitor, n (%) | 350 (98.0) | | 129 (97.7) | 221 (98.2) | 0.745 |
| β blockers, n (%) | 247 (69.2) | | 90 (68.2) | 157 (69.8) | 0.753 |
| ACEI/ARB, n (%) | 199 (55.7) | | 74 (56.1) | 125 (55.6) | 0.926 |
| Statins, n (%) | 334 (93.6) | | 119 (90.2) | 215 (95.6) | 0.045 |
| oral hypoglycemic drugs, n (%) | 87 (24.4) | | 25 (18.9) | 62 (27.6) | 0.067 |
| Insulin, n (%) | 44 (12.3) | | 11 (8.3) | 33 (14.7) | 0.079 |

p values < 0.05 indicate significance. BMI: body-mass index; PCI: percutaneous coronary intervention; CABG: coronary artery bypass grafting; HbA1c: glycated hemoglobin A1c; SHR: stress hyperglycemia ratio; CKMB: creatine kinase-myocardial band; BNP: brain natriuretic peptide; eGFR: estimated glomerular filtration rate; HDL: high-density lipoprotein; LDL: low-density lipoprotein; CRP: C-reactive-protein; LAD: left anterior descending; LCX: left circumflex artery; RCA: right coronary artery; TIMI: thrombolysis in myocardial infarction; PCI: percutaneous coronary intervention; ACEI: angiotensin converting enzyme inhibitor; ARB: angiotensin receptor blocker.
