# Supplementary material for: Association of stress hyperglycemia ratio with left ventricular function and microvascular obstruction in patients with ST-segment elevation myocardial infarction: a 3.0 T cardiac magnetic resonance study
Source: Cardiovasc Diabetol. 2024 May 27;23:179. doi: 10.1186/s12933-024-02271-6 (PMC11131267; doi:10.1186/s12933-024-02271-6)
Supplement: Supplementary file 2 — Supplementary Material 2. [file 12933_2024_2271_MOESM2_ESM.docx]

CMR Characteristics of the Study Population by Diabetes Status

| Variables | Overall  (n=357) | Diabetes  (n=132) | No Diabetes  (n=225) | P Value |
| --- | --- | --- | --- | --- |
| LVEF, % | 48.7±12.3 | 47.0±11.8 | 49.7±12.6 | **0.048** |
| LVGFI | 28.0±8.8 | 26.5±8.3 | 28.9±9.0 | **0.013** |
| LVEDV, ml | 130.0 (103.0,156.4) | 130.2 (97.4,157.6) | 129.9 (107.4,155.8) | 0.545 |
| LVESV, ml | 65.9 (47.6,87.4) | 68.2 (47.0,89.9) | 64.8 (47.8,86.0) | 0.724 |
| LVSV, ml | 60.4 (48.2,74.4) | 59.5 (46.8,70.9) | 61.8 (48.8,77.2) | 0.060 |
| CO, l/min | 4.4 (3.5,5.3) | 4.3 (3.4,5.1) | 4.4 (3.5,5.5) | 0.156 |
| LV-MASS, g | 132.4 (111.2,155.4) | 133.5 (110.2,157.8) | 132.4 (111.4,153.6) | 0.551 |
| GRS, % | 12.3±3.4 | 11.4±3.5 | 12.8±3.2 | **＜0.001** |
| GCS, % | -18.6±6.5 | -16.9±6.5 | -19.6±6.4 | **0.001** |
| GLS, % | -10.0±3.4 | -9.3±3.5 | -10.5±3.2 | **0.003** |
| Infarct size, (% LV mass) | 28.0 (18.5,37.3) | 25.8 (17.4,36.4) | 28.7 (18.6,37.7) | 0.269 |
| Extent of MVO, (% LV mass) | 1.0 (0.0,3.5) | 1.1 (0.0,4.6) | 1.0 (0.0,3.1) | 0.204 |
| Presence of MVO,  n (%) | 232 (65.0) | 89 (67.4) | 143 (63.6) | 0.459 |
| Presence of IMH, n (%) | 206 (57.7) | 81 (61.4) | 125 (55.6) | 0.284 |
| Location anterior, n (%) | 158 (44.3) | 62 (47.0) | 96 (42.7) | 0.429 |

p values < 0.05 indicate significance. CMR: cardiac magnetic resonance; SHR: stress hyperglycemia ratio; LVEF: left ventricular ejection fraction; LVGFI: left ventricular global function index; LVEDV: left ventricular end diastolic volume; LVESV: left ventricular end systolic volume; LV: left ventricular; SV: stroke volume; CO: cardiac output ; GRS: global radial strain; GCS: global circumferential strain; GLS: global longitudinal strain; MVO: microvascular obstruction; IMH: intramyocardial hemorrhage.
