# Supplementary material for: Association of stress hyperglycemia ratio with left ventricular function and microvascular obstruction in patients with ST-segment elevation myocardial infarction: a 3.0 T cardiac magnetic resonance study
Source: Cardiovasc Diabetol. 2024 May 27;23:179. doi: 10.1186/s12933-024-02271-6 (PMC11131267; doi:10.1186/s12933-024-02271-6)
Supplement: Supplementary file 3 — Supplementary Material 3. [file 12933_2024_2271_MOESM3_ESM.docx]

Associations of Fasting SHR grouping with Left Ventricular Function and Strain

|  |  | LVEF |  |  | LVGFI |  |  | GRS |  |  | GCS |  |  | GLS |  |  |
| --- | --- | --- | --- | --- | --- | --- | --- | --- | --- | --- | --- | --- | --- | --- | --- | --- |
|  |  | Beta Coefficient | Standard Error | P Value | Beta Coefficient | Standard Error | P Value | Beta Coefficient | Standard Error | P Value | Beta Coefficient | Standard Error | P Value | Beta Coefficient | Standard Error | P Value |
| Crude | SHR1 | Reference |  |  | Reference |  |  | Reference |  |  | Reference |  |  | Reference |  |  |
|  | SHR2 | -5.177 | 1.551 | 0.001 | -3. 150 | 1.106 | 0.005 | -0.598 | 0.426 | 0.161 | 1.438 | 0.827 | 0.083 | 0.608 | 0.425 | 0.153 |
|  | SHR3 | -8.139 | 1.531 | ＜0.001 | -5.687 | 1.092 | ＜0.001 | -2.138 | 0.420 | ＜0.001 | 3.779 | 0.816 | ＜0.001 | 2.068 | 0.419 | ＜0.001 |
| Model 1 | SHR1 | Reference |  |  | Reference |  |  | Reference |  |  | Reference |  |  | Reference |  |  |
|  | SHR2 | -4.965 | 1.449 | 0.001 | -2.949 | 1.009 | 0.004 | 0.560 | 0.377 | 0.139 | 1.265 | 0.722 | 0.080 | 0.497 | 0.364 | 0.173 |
|  | SHR3 | -5.881 | 1.468 | c | -4.011 | 1.023 | ＜0.001 | -1.273 | 0.382 | 0.001 | 2.102 | 0.731 | 0.004 | 1.197 | 0.369 | 0.001 |
| Model 2 | SHR1 | Reference |  |  | Reference |  |  | Reference |  |  | Reference |  |  | Reference |  |  |
|  | SHR2 | -3.790 | 1.322 | 0.004 | -2.184 | 0.948 | 0.022 | -0.240 | 0.345 | 0.487 | 0.615 | 0.655 | 0.349 | 0.273 | 0.346 | 0.431 |
|  | SHR3 | -4.605 | 1.350 | 0.001 | -3.196 | 0.969 | 0.001 | -0.938 | 0.353 | 0.008 | 1.414 | 0.670 | 0.035 | 0.988 | 0.354 | 0.006 |
| Model 3 | SHR1 | Reference |  |  | Reference |  |  | Reference |  |  | Reference |  |  | Reference |  |  |
|  | SHR2 | -3.446 | 1.265 | 0.007 | -1.982 | 0.887 | 0.026 | -0.128 | 0.316 | 0.686 | 0.430 | 0.613 | 0.489 | 0.191 | 0.329 | 0.563 |
|  | SHR3 | -3.915 | 1.308 | 0.003 | -2.971 | 0.917 | 0.001 | -0.753 | 0.327 | 0.022 | 1.139 | 0.634 | 0.073 | 0.862 | 0.341 | 0.012 |

p values < 0.05 indicate significance. SHR: stress hyperglycemia ratio; SHR1: SHR＜0.85; SHR2: 0.85≤SHR＜1.01; SHR3: SHR≥1.01. Model 1: adjusted for Age, Gender, Heart rate, Diabetes, Killip class, Location of culprit lesion and TIMI flow grade 0/1 pre-PCI, Oral hypoglycemic drugs, Use insulin. Model 2: adjusted for model 1 covariates + HbA1c, CKMBmass, Myoglobin, BNP and High-sensitive CRP. Model 3: adjusted for model 2 covariates + LVMASS, Infarct size and extent of MVO.SHR: stress hyperglycemia ratio; LVEF: left ventricular ejection fraction; LVGFI: left ventricular global function index; GRS: global radial strain; GCS: global circumferential strain; GLS: global longitudinal strain; TIMI: thrombolysis in myocardial infarction; HbA1c: glycated hemoglobin A1c; CKMB: creatine kinase-myocardial band; BNP: brain natriuretic peptide; CRP: C-reactive-protein; MVO: microvascular obstruction.
