# Supplementary material for: Association of stress hyperglycemia ratio with left ventricular function and microvascular obstruction in patients with ST-segment elevation myocardial infarction: a 3.0 T cardiac magnetic resonance study
Source: Cardiovasc Diabetol. 2024 May 27;23:179. doi: 10.1186/s12933-024-02271-6 (PMC11131267; doi:10.1186/s12933-024-02271-6)
Supplement: Supplementary file 4 — Supplementary Material 4. [file 12933_2024_2271_MOESM4_ESM.docx]

Univariable and Multivariable Logistic Regression Analysis of Fasting SHR Grouping and Presence of MVO in AMI

|  | Univariable Analysis in AMI | | Multivariable Analysis in AMI | |
| --- | --- | --- | --- | --- |
|  | OR (95% CI) | p value | OR (95% CI) | p value |
| Male, n (%) | 2.281 (1.259,4.135) | 0.007 | … | … |
| Heart rate, bpm | 1.025 (1.007,1.043) | 0.007 | … | … |
| SHR1 | Reference |  | Reference |  |
| SHR2 | 2.352 (1.387,3.987) | 0.001 | 1.509 (0.764,2.983) | 0.236 |
| SHR3 | 5.223 (2.913,9.367) | ＜0.001 | 3.878 (1.766,8.514) | 0.001 |
| CKMBmass, ng/ml | 1.009 (1.006,1.011) | ＜0.001 | 1.006 (1.003,1.009) | ＜0.001 |
| Myoglobin, ug/L | 1.001 (1.000,1.002) | 0.019 | … | … |
| BNP, pg/ml | 1.002 (1.001,1.003) | 0.003 | … | … |
| High-sensitive CRP, mg/L | 1.083 (1.043,1.125) | ＜0.001 | 1.073 (1.021,1.128) | 0.005 |
| TIMI flow grade 0/1 pre-PCI, n (%) | 0.248 (0.148,0.417) | ＜0.001 | 0.408 (0.203,0.817) | 0.011 |
| oral hypoglycemic drugs, n (%) | 0.508 (0.293,0.879) | 0.016 |  |  |
| LVEF, % | 0.927 (0.907,0.948) | ＜0.001 | 0.960 (0.933,0.988) | 0.006 |
| LV-MASS, g | 1.012 (1.005,1.019) | ＜0.001 | … | … |
| Infarct size, (% LV mass) | 1.115 (1.087,1.144) | ＜0.001 | 1.082 (1.050,1.114) | ＜0.001 |
| Location anterior, n (%) | 0.624 (0.400,0.974) | 0.038 | … | … |

p values < 0.05 indicate significance. SHR: stress hyperglycemia ratio; SHR1: SHR＜0.85; SHR2: 0.85≤SHR＜1.01; SHR3: SHR≥1.01. OR: odds ratio; CI: confidence interval; AMI: acute myocardial infarction; DM: Diabetes; CKMB: creatine kinase-myocardial band; BNP: brain natriuretic peptide; CRP: C-reactive-protein; TIMI: thrombolysis in myocardial infarction; PCI: percutaneous coronary intervention; LVEF: left ventricular ejection fraction
